# Biochemical and Nutritional Profiling of Pistachio Genotypes Grafted Onto *Pistacia khinjuk* Rootstocks

**DOI:** 10.1002/fsn3.71947

**Published:** 2026-05-27

**Authors:** Erdal Aglar, Burhan Ozturk, Cuneyt Uyak, Adnan Dogan, Onur Tekin, Orhan Durmaz, Davut Alan, Umut Ates

**Affiliations:** ^1^ Department of Landscape Architecture, Faculty of Arts, Design and Architecture Munzur University Tunceli Türkiye; ^2^ Department of Horticulture, Faculty of Agriculture Ordu University Ordu Türkiye; ^3^ Department of Horticulture, Faculty of Agriculture Van Yüzüncü Yıl University Van Türkiye; ^4^ Department of Horticulture, Faculty of Agriculture Sakarya Applied Sciences University Sakarya Türkiye

**Keywords:** mineral content, oil content, protein, yield

## Abstract

In the 2023 study, conducted in the Hizan District of Bitlis, aimed at determining the biochemical properties of pistachio trees grafted onto *Pistacia khinjuk* rootstocks, we identified 11 genotypes. Approximately 500 g of fruit were harvested from each genotype when the green fruit peel color started turning red, for use in the analyses. The various parameters were measured in the harvested fruits, including fruit size, yield, percentage of empty fruits, number of fruits per 100 g, splitting rate, moisture, ash, protein, fat, and mineral content. The fruit analyses revealed significant differences among the genotypes. The largest fruits were found in the G5 genotype, while the smallest were in the G9 genotype. Regarding yield, the highest ratio was observed in the G6 genotype with 46.63%, while the lowest was in the G10 genotype with 13.44%. The splitting rate, which varied by genotype, ranged between 0.97% and 41.88%. No statistical differences were observed in moisture and ash content among the genotypes, but protein content ranged from 18.01% to 28.49%, with the highest protein percentage recorded in the G9 genotype at 28.49%. Nutrient element analysis identified nitrogen (N), magnesium (Mg), calcium (Ca), and potassium (K) in the fruits. K emerged as the most abundant nutrient element across genotypes, followed by CA, magnesium (Mg), and sodium (Na). The G3 genotype exhibited the highest levels of potassium, calcium, magnesium, and manganese. In the fatty acid analyses, 9‐octadecenoic acid was found to be the most abundant fatty acid, ranging between 40.5% and 66.7% across genotypes. Additionally, other fatty acids such as hexadecanoic acid, 9,12‐octadecadienoic acid, and eicosanoic acid were also detected. These results indicated significant differences in the fatty acid content of the genotypes. As a result the pistachio genotypes examined in this study showed notable differences in terms of fruit yield, quality characteristics, and nutritional values. Particularly, G6 and G9 genotypes stood out with high yield and superior fruit qualities.

## Graphical abstract

This study was conducted to evaluate the pomological, biochemical, and nutritional characteristics of pistachio genotypes grafted onto *Pistacia khinjuk* rootstock in the Hizan region of Bitlis province. In the study, fruit size, kernel yield, cracking rate, moisture content, ash, protein, fat content, mineral content, and fatty acid composition were examined in 11 different genotypes. Significant differences were found among the genotypes in terms of fruit quality characteristics; G5 stood out in terms of fruit size, G6 in high kernel yield and cracking rate, G9 in protein content, and G3 in fat content, oleic acid, and mineral richness. Oleic acid was identified as the main component in the fatty acid profile, while potassium was the most dominant element in mineral content. PCA and dendrogram analyses showed that the genotypes were divided into different groups in terms of quality, nutritional value, and biochemical characteristics. The findings indicate that pistachio genotypes grown under Hizan's microclimate conditions have significant potential in terms of quality and nutritional value, and that genotype selection should be made in line with production objectives.

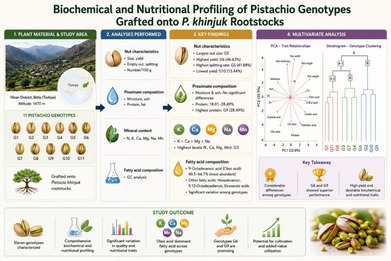

**Abbreviations**
Al: aluminium
Ca: calcium
K: potassium
Mg: magnesium
Mn: manganese
N: nitrogen

## 1 Introduction

Türkiye is a region that allows the growth of many cultivated fruit species in the world due to its different climate characteristics. Due to these properties, it has an important place in terms of world fruit cultivation. Türkiye, which is the region where many fruit species were first cultivated, is also among the gene centers of pistachios. Pistachios were first cultivated by Etiler in Southern Anatolia. Pistachios, which are among the indispensable items of kings' tables, are defined as the “golden tree” or “green gold” today because of their high price and being an important source of income (Kuru and Ozsabuncuoglu 1990). Türkiye has suitable ecological characteristics for pistachio cultivation and this species provides high returns to the grower, making it the third country in the world after Iran and the USA in terms of production. Although pistachio cultivation in Türkiye has become widespread in the Southeastern Anatolia region, the cultivation is carried out in many provinces using the rootstock potential of wild species of the *Pistacia genus* ( *P. terebinthus* L, *Pistacia khinjuk*, *P. atlantica, P. lentiscus*) widespread to different regions of our country. These wild species are important in terms of pistachio cultivation due to both the widespread cultivation in different areas and the positive effect on the yield potential of the variety on them. With the project of the Ministry of Agriculture and Forestry on grafting wild pistachios, many trees belonging to wild Pistacia species have been converted into culture varieties throughout the country (Kaska 1995). Within the scope of the project carried out in the Hizan region, which allows the cultivation of pistachios with its microclimate properties in the Eastern Anatolia region where cold and continental climate prevails, cultivation is carried out by grafting Siirt, Uzun and Red pistachio varieties onto *P. khinjuk* and *P. terebinthus* L. rootstocks. However, no study has been conducted on the fruit quality characteristics of pistachios grown in the region, which are important in terms of marketing and consumption, and this situation is not specific to the Hizan region.

The studies on pistachios have generally been limited to the Southeastern Anatolia region (Aydeniz 1990; Tekin et al. 2001). The number of studies on the fruit quality characteristics of pistachios, which have been grafted onto wild species and brought into cultivation in many provinces of Türkiye, is almost non‐existent. The studies conducted by Ozturk and Atac (1982); Caglar (1994); Isfendiyaroglu et al. (2001), Satil (2003) and Aglar et al. (2020) are rare studies in this sense. The mountainous structure of the Hizan region, which has an altitude (1470 m) where many fruit species cannot grow, causes the formation of different climatic zones in the region, while the microclimate areas formed in the valleys allow the growth of most fruit species. In the Eastern Anatolia region, where cold and continental climate prevails, pistachio cultivation in the Hizan region, which allows the growth of fruit species such as chestnut, hazelnut, pomegranate, and fig with its microclimate feature, is an important and valuable potential. With the study we have prepared in order to reveal this potential, the pomological and biochemical properties of the fruit were determined in the trees of the Siirt pistachio variety grafted onto *P. khinjuk* rootstock in the villages of Göktepe, Sağırkaya, Döküktaş, Nuh and Hacımehmet in the Hizan district of Bittlis.

## 2 Material and Method

### 2.1 Plant Material

This study was carried out in the villages of Göktepe, Sağırkaya, Döküktaş, Nuh and Hacımehmet in Hizan District of Bittlis. The population of Siirt pistachio varieties grafted onto *P. khinjuk* rootstock in the region was viewed and a sufficient number of trees suitable for the purpose of the study were determined within the knowledge of the grower, taking into account the fruit yield and quality characteristics. Measurements and analyses were carried out to determine the pomological and biochemical properties of the fruits harvested from these trees.

### 2.2 Method

In order to make fruit analyses and measurements, 150 fruit were randomly taken from different parts of each tree to represent the whole tree. The fruits were separated from their green shells, placed in special plastic packages that were ventilated and labeled. After the fruits dried naturally, the following pomological measurements and analyses were made.

### 2.3 Fruit Width, Fruit Length and Fruit Height

The widest part of the equatorial part of the fruit is the fruit width, the narrowest part is the fruit height and the fruit length between the two poles representing the stem pit and the nose region of the fruit was measured with the help of a digital caliper with a precision of 0.01 mm and determined in 25 fruit samples.

### 2.4 Fruit Weight

In the fruits taken as a sample, the weight of 100 grains as peeled and inner fruit was determined in grams by weighing them on a precision scale with a sensitivity of 0.001 g.

### 2.5 Efficiency

It was determined as the weight of the inner amount obtained from 100 g of peeled fruit.

Efficiency = (Inner fruit weight/Shelled fruit weight) × 100 was calculated.

### 2.6 Empty (%) Fruit Ratios

A total of 12 bunches of fruit, consisting of the first three bunches formed on the shoots, were taken from each tree in all directions (East, West, South and North) and the full (%) and empty (%) fruit ratios were determined.

### 2.7 The Number of Fruits in 100 g

It was determined by weighing 100 g of fruit on a 0.01‐g precision scale and counting the fruits in 100 g.

### 2.8 Cracking Ratio

The number of cracked fruits was counted in a sufficient number of fruit samples taken from the trees and calculated as a percentage.

### 2.9 Double‐Inner Ratio

The number of double‐inner fruits was determined in a sufficient number of fruit samples taken from the trees and calculated as a percentage.

### 2.10 Moisture and Ash Ratio

The fruits were ground and placed in an oven set at 105°C for 4 h with three repetitions and 3 g for each repetition. After this period, the samples taken from the oven were put in a desiccator and kept for 45 min, then weighed, and the percentage moisture content of the samples was determined. Ash determination in fruits was carried out according to Anonymous (1982). Three grams of ground sample was placed in the tared crucibles for each repetition, 2–3 drops of ethanol were dropped on it, and pre‐burning was performed. After the pre‐washing process, the ground samples were placed in a 500°C muffle furnace, and after the complete washing process was completed, the samples were taken out of the muffle furnace and kept in a desiccator for 45 min, their weights were weighed, and ash determinations were made based on dry weight.

Ash amount based on wet weight = [(Crucible + sample weight) − (crucible weight)/Weight of weighed powder sample] × 100.

Ash amount based on dry weight = (Ash amount based on wet weight/100 − % moisture) × 100 formulas were calculated.

### 2.11 Protein Ratio

The protein amounts of fruit were determined using the Kjeldahl method Bremner (1965). In this method, which is essentially a wet combustion method, the nitrogen in the samples is converted to ammonium (NH_4_) by burning with concentrated H_2_SO_4_ and the nitrogen is determined from the amount of NH_3_ found as a result of the titration of the ammonia (NH_3_) released as a result of distillation in an alkaline environment (Kacar 1972). 0.25 g of ground sample was taken from each replicate and placed on tissue papers and placed in tubes. For the pre‐combustion process, 1 spatula of the accelerator catalyst salt mixture was added to each Kjeldahl tube and 5 mL of salicylic‐sulfuric acid was added to ensure that the tissue paper reacted completely with the acid. After the overnight pre‐digestion process, the samples were taken to the ‘Kjeldatherm Gerhardt’ digestion unit and kept at the temperatures of 70°C, 170°C and 270°C for 1 h and at 370°C for 5 h, respectively. After the digestion process, distillation was performed in the “Gerhardt Vapodest” device. In this process, 10 mL of pure water and 20 mL of NaOH were added to the samples, the medium became alkaline, ammonium turned into ammonia and reacted with boric acid to form ammonium borate (Kacar 1972). After this process, which lasted approximately 2–3 min, a titration was performed with 0.37 N H_2_SO_4_ to determine the % N value. The nitrogen (N) value was multiplied by the coefficient of 5.30 determined for pistachios and the % protein amount was determined (USDA 2010).

### 2.12 Oil Ratio

The Soxhelet extraction method was used to determine the total oil content in fruits. For each replicate, 10 g of ground sample was taken and placed in cartridges and lightly covered with cotton. The cartridges were placed in the Soxhelet device and 150 mL of n‐hexane was added to them. After the extraction process at 100°C–105°C for 6 h, the oil and hexane in the flask were subjected to nitrogen gas and the hexane was evaporated, and the flask and the oil in it were weighed together and the amount of oil in the sample was determined (Ayfer 1973).

### 2.13 Mineral Substance Ratio

The amount of mineral substance in fruits was determined by the wet combustion method (Kacar 1972). For each replication, 5 g of chopped fruit samples were kept in an oven set at 65°C for 48 h to determine the moisture content. The samples were placed in an Erlenmeyer flask and 10 mL of nitric acid: perchloric acid at a ratio of 4:1 was added to them and the Erlenmeyer flask was placed on a hot plate. The temperature was slowly increased to 150°C–200°C. The combustion was continued until the dense white fumes of perchloric acid completely covered the inside of the Erlenmeyer flask. After the combustion process was completed, the samples were left to cool and poured into 50 mL volumetric flasks with a glass funnel and made up to 50 mL with pure water. The samples were filtered through a “Whatman 42” filter paper and stored in storage containers and kept at refrigerator temperature to be read. In order to read the mineral substances, a dilution of 1/10 mL was made. Na and K minerals were determined with a Jenway PFP 7 brand flame photometer, P mineral was determined with a Shimadzu UV‐160 brand spectrophotometer according to the vanadomolybdenum phosphoric yellow color method, and Ca, Mg, Fe, Mn, Cu and Zn were determined with a Varian 720‐ES ICP (Inductively coupled plasma) Optical Emission Spectrometer (Kacar 1972).

### 2.14 Statistical Analysis

The data, whose normal distribution suitability was determined by the Kolmogorov‐Simirnov test and whose homogeneity was checked by the Levene test, were evaluated by analysis of variance. The significance level between applications was determined by Tukey's multiple comparison test. The significance level in interpreting the statistical results performed with the SAS package program (SAS 9.1 version, USA) is *α* = 5%. PCA analysis, which explains the relationship between genotypes and examined traits, was performed using the JMP Pro 17 statistical package program.

## 3 Results

### 3.1 Fruit Size and Yield

Fruit size, which is considered as fruit weight, fruit width, fruit length, fruit height and internal weight, was significantly different among the genotypes. While the G5 genotype had the largest fruits (138.20 g/100 fruits), the smallest fruits (69.70 g/100 fruits) were harvested in the G9 genotype. Fruit height, length and width varied between 9.22 (G9) and 11.92 (G5), 21.53 (G1) and 19.10 (G9) and 10.82 (G6) and 12.75 (G5), respectively (Table 1). The number of fruits per 100 g in genotypes varies between 95 (G4) and 137 (G6). While the highest value in terms of yield ratio showing the internal yield in pistachio was obtained in G6 genotype (46.63%), this genotype has a significantly higher internal yield than the others. The lowest yield ratio is seen in G10 genotype (13.44%). In general, G3 (41.20%) and G11 (34.88%) genotypes also have high yield ratios. Empty fruit ratio was determined as 0.00% in all genotypes, which shows that all genotypes evaluated in the study did not produce empty fruit. Double kernel ratio was determined as 0.00% in all genotypes, that is, double kernel (the presence of two pistachios in a single shell) was not observed. The G6 genotype stands out with both the highest yield (46.63%) and the highest cracking rate (41.88%) (Table 2).

**TABLE 1:** Nut characteristics of pistachio ( *Pistacia vera* *L.*) genotypes from Hizan region.

Genotype	Shell width (mm)	Shell length (mm)	Shell thickness (mm)	Weight of 100 shell (g)
G1	12.60 ± 0.18a	21.53 ± 0.37ab	11.56 ± 0.22a	92.40 ± 9.76b–e
G2	11.41 ± 0.17bc	19.88 ± 0.30cd	10.44 ± 0.24b	78.60 ± 5.46de
G3	11.80 ± 0.15a–c	20.31 ± 0.33bd	10.48 ± 0.18b	104.80 ± 5.12b
G4	11.72 ± 0.21a–c	20.45 ± 0.25bd	10.64 ± 0.17b	103.30 ± 6.55bc
G5	12.73 ± 0.28a	22.29 ± 0.54a	11.92 ± 0.35a	138.20 ± 10.49a
G6	10.82 ± 0.21c	19.47 ± 0.37cd	9.30 ± 0.23c	83.40 ± 4.21b–e
G7	11.78 ± 0.19a–c	20.76 ± 0.30bc	10.86 ± 0.22b	98.20 ± 7.21b–d
G8	12.09 ± 0.28ab	20.87 ± 0.41bc	10.83 ± 0.29b	87.60 ± 10.30b–e
G9	11.05 ± 0.08c	19.10 ± 0.22d	9.22 ± 0.24c	69.70 ± 3.27e
G10	11.71 ± 0.16a–c	19.86 ± 1.16cd	10.85 ± 0.15b	77.40 ± 4.66de
G11	12.29 ± 0.86ab	20.83 ± 0.20bc	10.70 ± 0.15b	81.20 ± 6.19c–e

*Note:* Means in columns with the same letter do not differ according to Tukey's test at *p* < 0.05.

**TABLE 2:** Nut number, kernel ratio, empty nut, double kernel ratio and split suture ratio in Hizan region pistachio ( *Pistacia vera* *L.*) genotypes.

Genotype	Shell number in 100 g	Kernel ratio (%)	Empty shell (%)	Cracking ratio (%)	Double kernel ratio (%)
G1	107.21 ± 3.21c	21.78 ± 1.15c	0.00 ± 0.00a	27.91 ± 3.21c	0.00 ± 0.00a
G2	125.24 ± 2.08b	20.85 ± 2.08b	0.00 ± 0.00a	33.80 ± 2.31b	0.00 ± 0.00a
G3	99.90 ± 0.58d	41.20 ± 1.73d	0.00 ± 0.00a	21.25 ± 1.73d	0.00 ± 0.00a
G4	95.24 ± 2.31d	32.14 ± 1.53bc	0.00 ± 0.00a	29.60 ± 1.53bc	0.00 ± 0.00a
G5	96.06 ± 2.08d	25.54 ± 1.15f	0.00 ± 0.00a	0.97 ± 0.00f	0.00 ± 0.00a
G6	136.59 ± 2.52a	46.63 ± 2.08a	0.00 ± 0.00a	41.88 ± 1.15a	0.00 ± 0.00a
G7	108.27 ± 2.65c	32.15 ± 2.52f	0.00 ± 0.00a	0.00 ± 0.00f	0.00 ± 0.00a
G8	97.66 ± 2.08d	30.46 ± 1.53f	0.00 ± 0.00a	1.98 ± 0.58f	0.00 ± 0.00a
G9	133.33 ± 1.73a	27.81 ± 2.08f	0.00 ± 0.00a	1.20 ± 0.58f	0.00 ± 0.00a
G10	134.36 ± 2.08a	13.44 ± 1.73e	0.00 ± 0.00a	8.65 ± 1.73e	0.00 ± 0.00a
G11	111.32 ± 3.79c	34.88 ± 1.53e	0.00 ± 0.00a	10.28 ± 2.52e	0.00 ± 0.00a

*Note:* Means in columns with the same letter do not differ according to Tukey's test at *p* < 0.05.

### 3.2 Moisture and Ash Ratio

Since pistachios with lower moisture content can generally last longer, no statistically significant difference was detected between the genotypes in terms of fruit moisture content, which is an important criterion in terms of storage conditions and durability. The moisture content of the genotypes varied between 9.10% and 12.55%. While the highest moisture content was determined in the G9 (12.55%) genotype, the lowest moisture content was recorded in the G6 (9.10%) genotype. Ash content indicates the mineral content of the fruit and there was no significant difference between the genotypes. Ash content varied between 2.13% and 3.85%. While the highest ash content was recorded in the G3 (3.85%) genotype, the lowest ash content was detected in the G2 (2.13%) and G6 (2.15%) genotypes. Genotypes with higher ash content may have higher mineral content. Protein content showed significant differences between the genotypes. Protein content varied between 18.01% and 28.49%. The highest protein ratio was obtained in the G9 (28.49%) genotype, while the lowest protein ratio was determined in the G6 genotype (18.01%). G9 stands out with the highest protein ratio (28.49%) and the highest moisture ratio (12.55%). While oil ratio varies depending on the genotype, the highest value (51.80%) was recorded in the G3 genotype, while the lowest ratio was obtained with the G10 genotype (Table 3).

**TABLE 3:** Moisture, ash, protein and oil ratio in pistachio ( *Pistacia vera* *L.*) genotypes from Hizan region.

Genotype	Moisture (%)	Ash (%)	Protein (%)	Oil ratio (%)
G1	12.35 ± 1.15a	3.58 ± 1.53a	22.01 ± 1.73b–e	47.20 ± 1.63bc
G2	10.36 ± 1.53a	2.13 ± 1.15a	19.11 ± 1.73de	48.9 ± 1.72b
G3	10.25 ± 1.53a	3.85 ± 1.15a	23.81 ± 1.53a–d	51.80 ± 1.53a
G4	10.11 ± 2.31a	2.61 ± 0.58a	23.11 ± 1.15a–e	50.12 ± 2.16ab
G5	12.30 ± 1.73a	3.09 ± 1.15a	19.77 ± 1.15c–e	45.09 ± 1.93c
G6	9.10 ± 2.52a	2.15 ± 0.58a	18.01 ± 2.31e	46.89 ± 1.60bc
G7	10.21 ± 1.73a	2.47 ± 1.15a	26.93 ± 2.52ab	49.01 ± 1.57b
G8	11.98 ± 2.08a	3.26 ± 1.53a	24.89 ± 1.53a–c	47.33 ± 2.73bc
G9	12.55 ± 0.58a	3.10 ± 0.58a	28.49 ± 1.15a	51.09 ± 1.48a
G10	12.15 ± 0.58a	2.99 ± 1.53a	22.63 ± 2.08b–e	45.20 ± 1.19c
G11	12.32 ± 1.15a	3.76 ± 1.53a	21.06 ± 1.15c–e	46.01 ± 1.90c

*Note:* Means in columns with the same letter do not differ according to Tukey's test at *p* < 0.05.

### 3.3 Oil Ratio

Fatty acids such as palmitic acid, palmitoleic acid, oleic acid, linoleic acid, linolenic acid, arachidic acid, gondolaic acid, and pelargonic acid were detected in pistachio fruits. The most abundant fatty acid in the study, oleic acid, varied between 40.5% (G10) and 66.7% (G3). While the amount of palmitoleic acid varied between 0.44% and 1.20%, the highest value was recorded in the G10 genotype. It was found that the amounts of palmitic acid, oleic acid, arachidic acid, and gondolaic acid varied between 17.9% (G8) and 29.7% (G9), 10.1% (G8) and 21.2% (G10), 0.33% (G8) and 0.50% (G7), and 0.28% (G10) and 0.60% (G3), respectively. While linolenic acid was not detected in the G1, G3, G4, G5, G6, G8, and G11 genotypes, it was detected as 0.22%, 0.39%, 0.43%, and 0.58% in the G2, G7, G9, and G10 genotypes, respectively. While 7.39% pelargonic acid was recorded with the G10 genotype, it was not detected in the other genotypes (Table 4).

**TABLE 4:** Oil acid content in pistachio ( *Pistacia vera* *L.*) genotypes from Hizan region.

Oil acid content (%)	Genotypes										
	1	2	3	4	5	6	7	8	9	10	11
Palmitic acid	21.2	22.9	17.9	23.6	20.1	20.4	23.2	19.0	29.7	28.4	26.1
Palmitoleic acid	1.07	1.12	0.44	0.60	0.58	0.58	0.62	0.45	0.91	1.20	0.99
Oleic acid	58.7	55.2	66.7	64.2	66.2	63.7	58.0	50.7	53.5	40.5	55.8
Linoleic acid	18.1	19.8	13.9	10.6	12.2	14.4	16.7	10.1	14.6	21.2	16.4
Linolenic acid	nd	0.22	nd	nd	nd	nd	0.39	nd	0.43	0.58	nd
Arachidic acid	0.38	0.41	0.48	0.47	0.46	0.45	0.50	0.33	0.46	0.44	0.47
Gondoic acid	0.37	0.37	0.60	0.44	0.47	0.53	0.58	0.37	0.34	0.28	0.31
Pelargonic acid	nd	nd	nd	nd	nd	nd	nd	nd	nd	7.39	nd

Abbreviation: nd, not determined.

### 3.4 Mineral Substance Ratio

As a result of the analysis, nutrients such as N, Mg, Ca, K, Na, Cu, Mn, Al, Fe, and Zn were detected in the fruit. While nitrogen was determined between 2.88% and 4.56% in pistachio fruit, no significant difference occurred between the genotypes. When other plant nutrients were compared, the plant had the highest amount of K (between 997.37 and 1848.67 mg kg^−1^), followed by Ca (between 231.82 and 1449.09 mg kg^−1^), Mg (between 119.97 and 708.99 mg kg^−1^), Na (between 83.58 and 374.67 mg kg^−1^), Fe (between 0.70 and 45.53 mg kg^−1^), Zn (between 1.89 and 7.90 mg kg^−1^), Al (between 0.11 and 5.73 mg kg^−1^), Cu (between 0.83 and 5.34 mg kg^−1^), and Mn (between 0.38 and 3.76 mg kg^−1^). The content of nutrient elements varied depending on the genotype. It was determined that the G3 genotype had the highest values in terms of K, Ca, Mg, and Mn contents. While higher values were obtained with the G7 genotype in terms of Fe and Zn, it was determined that G6 had richer content in terms of Na, G5 in terms of Al, and G10 in terms of Cu (Table 5).

**TABLE 5:** Nutrient elements in pistachio ( *Pistacia vera* *L.*) genotypes from Hizan region (mg kg^−1^).

Genotype	*N* (%)	Mg	Ca	K	Na
G1	3.52 ± 0.58a	329.07 ± 4.16e	600.09 ± 4.16f	1502.50 ± 4.16e	162.64 ± 2.31i
G2	3.06 ± 1.53a	158.88 ± 2.52j	231.82 ± 1.53j	1024.64 ± 1.53i	83.58 ± 2.08j
G3	3.81 ± 0.58a	708.99 ± 2.31a	1449.09 ± 2.31a	1848.67 ± 3.06a	356.78 ± 3.46c
G4	3.70 ± 1.53a	270.71 ± 3.06 g	646.92 ± 2.31e	1452.16 ± 3.06f	365.75 ± 3.79b
G5	3.16 ± 1.15a	429.25 ± 1.53d	772.22 ± 6.11d	1620.89 ± 4.04d	313.96 ± 1.53d
G6	2.88 ± 1.15a	295.28 ± 3.06f	814.55 ± 2.31c	1408.96 ± 3.06 g	374.67 ± 2.31a
G7	4.31 ± 1.73a	599.43 ± 1.73b	976.09 ± 2.08b	1800.38 ± 2.31b	316.94 ± 2.31d
G8	3.98 ± 0.58a	119.97 ± 3.06 k	262.35 ± 1.15i	997.37 ± 2.65 j	235.36 ± 2.89f
G9	4.56 ± 1.15a	230.63 ± 2.31 h	569.29 ± 1.15 g	1665.95 ± 2.08c	205.88 ± 2.08 h
G10	3.62 ± 1.15a	214.45 ± 7.77i	478.31 ± 2.00 h	1353.67 ± 2.89 h	222.64 ± 2.31 g
G11	3.37 ± 1.53a	446.25 ± 3.61c	768.95 ± 3.79d	1674.02 ± 3.21c	277.44 ± 2.31e

Genotype	Cu	Mn	Al	Fe	Zn
G1	4.34 ± 1.15ab	2.07 ± 1.53ab	2.06 ± 0.58bc	14.60 ± 1.73ef	1.89 ± 0.58c
G2	1.84 ± 0.58ab	0.68 ± 0.03b	1.13 ± 0.03bc	12.36 ± 1.53f	1.98 ± 0.06c
G3	2.53 ± 0.00ab	3.76 ± 1.00a	2.28 ± 0.58bc	25.88 ± 2.08c	2.00 ± 0.57c
G4	3.79 ± 1.73ab	1.20 ± 1.00ab	0.17 ± 0.06c	1.54 ± 0.58 g	3.86 ± 0.88bc
G5	4.00 ± 1.52ab	2.29 ± 1.15ab	5.73 ± 1.73a	33.78 ± 1.53b	4.60 ± 1.73a–c
G6	1.70 ± 0.58b	2.29 ± 0.58ab	3.16 ± 2.08ab	18.92 ± 1.53de	6.17 ± 1.15ab
G7	0.83 ± 0.04ab	2.52 ± 0.46ab	2.81 ± 0.58a‐c	45.53 ± 2.65a	7.90 ± 1.53a
G8	3.30 ± 1.73ab	0.38 ± 0.03b	3.81 ± 1.15ab	40.95 ± 2.43a	2.21 ± 1.15c
G9	2.71 ± 1.15ab	1.62 ± 0.58ab	0.11 ± 0.00d	22.85 ± 1.73 cd	4.43 ± 0.53bc
G10	5.34 ± 1.15a	1.04 ± 0.58ab	3.17 ± 1.15a–c	0.70 ± 0.03 g	4.13 ± 1.15bc
G11	2.19 ± 1.15ab	3.71 ± 1.15a	2.10 ± 1.15bc	21.01 ± 1.73 cd	5.20 ± 1.15a–c

*Note:* Means in columns with the same letter do not differ according to Tukey's test at *p* < 0.05.

### 3.5 Principal Component Analysis

**FIGURE 1:**
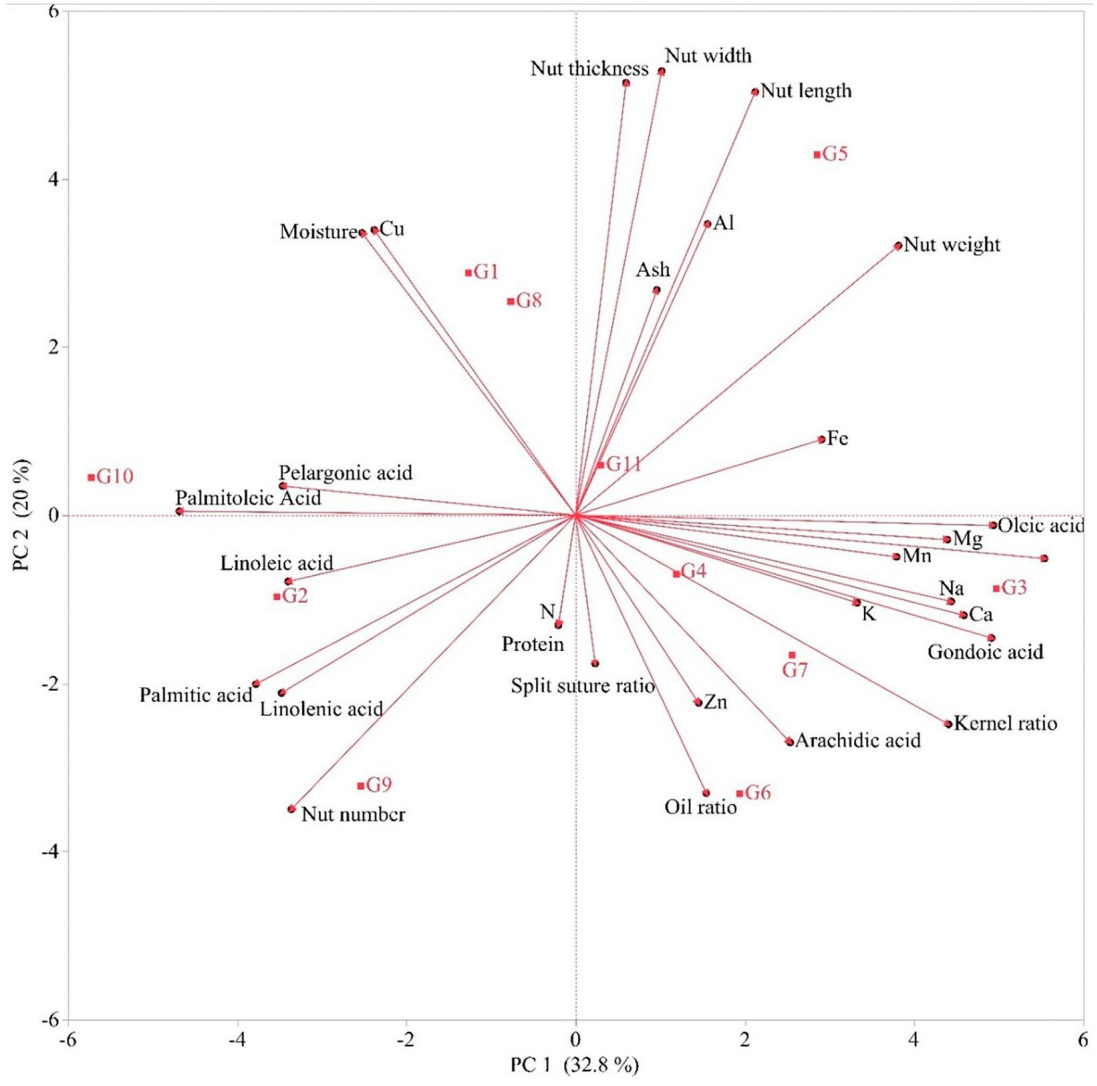
Characterization of Hizan region pistachio ( *Pistacia vera* L.) genotypes; nut characteristics, oil acid composition, nutritional contents.

For the Principal Component Analysis of pistachio, 30 characteristics were used. Out of 30 principal components, 6 PCs had eigenvalues > 1.0 and explained 89.96% of the total variation. In fact, the first component explained 32.84% of the cumulative variance, while the first two components explained 52.84% and the first three components explained 67.33%. In addition, the first four components explained 77.54%, the first five components explained 85.07% and the first six components explained 89.96%. PC1 (32.84% of the total variance) was mainly associated with nut weight, kernel weight, kernel ratio, Mg, Ca, Na, Mn, Fe, palmitic acid, palmitoleic acid, oleic acid, linoleic acid, linolenic acid, gondolaic acid and pelargonic acid. PC2, which explained 52.84% of the variability, was associated with nut width, nut length, nut thickness, nut number, moisture, oil ratio and Al. However, PC3, which explained 67.33% of the variability, was associated with split suture ratio, protein, N and K. In addition to all these, PC4, which explained 77.54% of the variability, was associated with arachidic acid, PC5, which explained 85.07%, was associated with ash and Zn and PC6, which explained 89.96%, was associated with Cu. When the main characteristics affecting the Principal Component Analysis were examined; PC1 kernel weight value (0.96), PC2 nut width (0.92), PC3 split suture ratio (−0.79), PC4 arachidic acid (0.49), PC5 Zn (−0.61) and PC6 Cu (0.60). In general, it was revealed that the characteristics in PC1 and PC2 of pistachio genotypes were highly related to each other with the Principal Component Analysis (Figure 1; Table 6).

**TABLE 6:** Principal component analysis of pistachio ( *Pistacia vera* L.) genotypes in Hizan region.

Characteristics	PC1	PC2	PC3	PC4	PC5	PC6
Nut width	0.17803	**0.92339**	0.21461	0.07973	0.14153	−0.10832
Nut length	0.37082	**0.87964**	0.06855	0.08099	−0.04938	−0.11233
Nut thickness	0.10426	**0.89865**	0.11923	0.17353	−0.00008	−0.00117
Nut weight	**0.66693**	0.56007	0.00089	0.12623	−0.12862	0.23869
Kernel weight	**0.96834**	−0.09014	−0.14936	−0.00204	−0.03512	0.10470
Nut number	−0.58676	**−0.61148**	−0.06136	0.33345	−0.14868	−0.12239
Kernel ratio	**0.77034**	−0.43478	−0.21114	−0.07869	−0.00615	−0.06362
Split suture ratio	0.04062	−0.30847	**−0.79768**	0.24636	0.39138	0.03722
Moisture	−0.44082	**0.58658**	0.51318	−0.10891	0.12321	−0.10172
Ash	0.16810	0.46843	0.48023	−0.07727	**0.54061**	−0.05172
Protein	−0.03501	−0.22650	**0.75074**	−0.58481	−0.00856	0.09507
Oil ratio	0.26996	**−0.57777**	0.13428	−0.50958	0.43682	0.06609
N	−0.03548	−0.22866	**0.75031**	−0.58456	−0.00721	0.09487
Mg	**0.76798**	−0.05096	0.43830	0.32036	0.17462	−0.13738
Ca	**0.80189**	−0.20815	0.32788	0.30061	0.22283	0.06717
K	0.58168	−0.18204	**0.65939**	0.31890	0.22269	0.00238
Na	**0.77577**	−0.17940	0.06255	0.16015	−0.18510	0.46657
Cu	−0.41574	0.59294	0.06562	0.09673	0.20487	**0.60763**
Mn	**0.66335**	−0.08678	0.34391	0.44875	0.31414	−0.26547
Al	0.27264	**0.60509**	−0.02719	0.26671	−0.59095	−0.03246
Fe	**0.50852**	0.15729	0.29850	−0.37862	−0.49668	−0.46160
Zn	0.25418	−0.39026	0.32924	0.36061	**−0.61175**	−0.05334
Palmitic acid	**−0.66002**	−0.35117	0.49344	0.16923	0.02784	0.09150
Palmitoleic acid	**−0.81779**	0.00811	0.10113	0.40200	0.27879	−0.24113
Oleic acid	**0.86280**	−0.02156	−0.28053	0.03464	0.21289	−0.00163
Linoleic acid	**−0.59359**	−0.13778	0.13478	0.58557	0.12485	−0.34269
Linolenic acid	**−0.60703**	−0.36949	0.53485	0.13466	−0.28602	0.08433
Arachidic acid	0.44344	−0.47264	0.42402	**0.49132**	0.01901	0.15737
Gondoic acid	**0.85876**	−0.25521	−0.06868	0.02843	−0.15474	0.02296
Pelargonic acid	**−0.60469**	0.06040	0.24027	0.43902	−0.15600	0.45325
Eigen value	9.8527	5.9999	4.3485	3.0613	2.2586	1.4677
% variance	32.842	20	14.495	10.204	7.529	4.892
Cumulative variance	32.842	52.842	67.337	77.541	85.07	89.962

*Note:* Factor loading ≥**49.1** are bold.

### 3.6 Dendrogram Analysis

**FIGURE 2:**
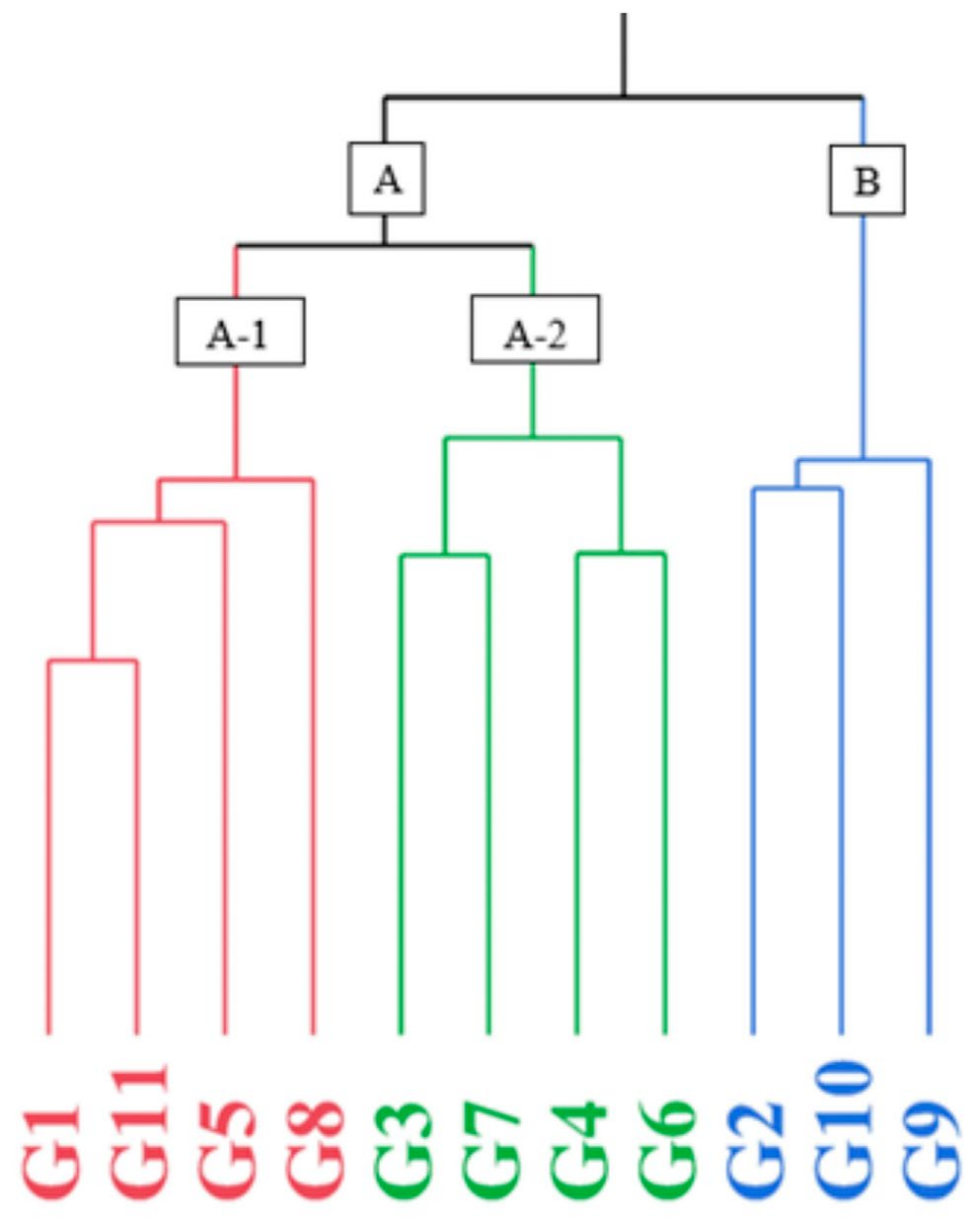
Dendrogram grouping of Hizan region pistachio ( *Pistacia vera* L.) genotypes; nut characteristics, oil acid composition, nutritional contents, and biochemical characteristics.

Dendrogram analysis was performed using a total of 30 characteristics including nut width, nut length, nut thickness, nut weight, kernel weight, nut number, kernel ratio, split suture ratio, moisture, ash, protein, oil ratio, N, Mg, Ca, K, Na, Cu, Mn, Al, Fe, Zn, palmitic acid, palmitoleic acid, oleic acid, linoleic acid, linolenic acid, arachidic acid, gondolaic acid and pelargonic acid of the examined pistachio genotypes. As a result of the dendrogram analysis, two main groups A and B emerged. In addition, group A was divided into two subgroups as A‐1 and A‐2. The first main group, group A, consisted of 8 genotypes. Four of the eight genotypes constituting group A (G1, G5, G8 and G11) represented group A‐1, and the other four (G3, G4, G6, and G7) represented group A‐2. The second main group B consists of three genotypes (G2, G9, and G10) (Figure 2).

## 4 Discussion

The same variety on different rootstocks shows significant differences in terms of development, yield, product quantity and quality, tree life and adaptation to ecological conditions, and rootstock and scion have important physiological effects on each other (Ozcagiran 1974). One of the most important properties of rootstocks is their contribution to the widespread of cultivation in species due to their ability to adapt to different climates. Pistachio is the best example in this sense. *P. khinjuk*, with its cold resistance property, allows pistachio cultivation in a cold region like Bitlis. Taking advantage of the microclimate property and rootstock characteristics of *P. khinjuk* species in its flora, pistachio cultivation is economically carried out in the village of Hizan district of Bitlis. In the study, the significant differences were determined among pistachio genotypes in terms of fruit size, weight and yield characteristics. The fruit weight varied between 69.70 and 138.20 g/100 fruit, and yield varied between 41.88% and 46.63%. When the studies conducted in this sense were examined, in the study where Uzun variety grafted on *Pistacia terebinthus* was used in the Çanakkale‐Ayvacık region, 100‐nut weight with shell was determined as 111 g and yield was determined as 41.88% (Satil 2003). In the study conducted in the Pervari district of Siirt between 2004 and 2005, fruit and tree characteristics of 43 genotypes selected from both pistachio varieties grown in the region and wild population were defined. In these genotypes, the average fruit weights were observed to be 1.21–1.93 g, internal weights were 0.46–0.81 g, and yields were between 31.5% and 49.0% (Ozturk 2006). In a study conducted in Gaziantep between 1984 and 1987, it was determined that 100‐seed weight was 133.82 g in the long variety, and yield was 40% (Karaca and Nizamoglu 1995). In the study conducted by Aglar et al. (2020) in Günlüce village of Suşehri district of Sivas in order to determine the fruit quality characteristics of Uzun variety grafted on *P. terebinthus* rootstock, they determined the fruit weight as 0.93 g and yield as 53.8%. When compared with the study results, it is seen that the fruits of pistachio grown in Hizan are relatively small but yield is high. The use of pollinators, variety, climate conditions, rootstock and cultural practices have an important effect on the formation of empty fruits (Crane and Iwakiri 1980). Crane (1975) reported that the formation of empty fruits in the Kerman variety grafted on *P. vera* seedlings was due to seed abortion and parthenocarpy, and the empty fruit rate was 26%. In a 4‐year study on the Siirt variety, the empty fruit rate was found to be 13.05% (Ak 1997). In the study, the empty fruit rate was determined as 0% in all genotypes and no double internal situation was observed. This result may be thought to be due to the higher fertilization rate when a pollinator variety is grafted onto one branch of each tree during grafting in the region, as well as rootstock and climate characteristics. Caglar (1994) reported that empty internal formation is due to lack of pollination, competition between fruits, and lack of cultural practices such as irrigation and fertilization. Goldhamer et al. (1987) suggested that rootstocks are also effective in empty fruit formation. The cracking in pistachios is one of the most important characteristics affecting quality and marketing. In addition to factors such as variety, age of the tree, nutritional status, temperature difference between day and night during ripening (Ak 1997), genetic variation among rootstocks is also effective in cracking (Goldhamer et al. 1987). In the study, the cracking rate was determined as 0.97% and 41.88% in genotypes, while in similar studies, cracking rate was determined as 46% (Caglar 1994), 67.2% (Tekin and Akkok 1995), 61% (Yildiz 1999) and 80% (Aglar et al. 2020) in long variety. When compared with the study results, it was observed that the cracking rate was low in pistachios grown in Hizan. I believe that this low cracking rate was due to the early harvest due to different concerns in the region and therefore the cracking did not occur completely in the fruits. Appropriate moisture content (4%–5% w/w) in dried pistachios is an important factor for quality (Kashani et al. 2003). Although moisture content between 4% and 6% produces positive differences in some sensory properties (Kader et al. 1982), it is impossible to reduce moisture exactly to 5% by commercial drying. In the study, it was observed that the humidity rates of the genotypes varied between 9.10% and 12.55% and were high compared to other studies. In fact, Aglar et al. (2020) determined the humidity rate as 3.73% in the study they conducted in Günlüce village of Suşehri district of Sivas in order to determine the fruit quality characteristics of Uzun variety grafted onto *P. terebinthus* (melengiç) rootstock. Again, Tsantili et al. (2011) reported the humidity rate as between 4.43% (Mumtaz) and 6.05% (Kerman) in their study on different varieties. In the study, the protein ratio, which varies depending on the genotypes, varies between 18.01% and 28.49%. When similar studies are examined, Seferoglu et al. (2006) found that the protein amount in their study with the long variety in 7 different regions of the Büyük Menderes basin was 22.6%–32.0%; Tsantili et al. (2011) found that the protein amount in their study on different varieties varied between 19% d.w. (Joley) and 21.8% d.w. (Cerasola). Again, Aglar et al. (2020) determined the protein content as 19.39% in the study they conducted in Günlüce village of Suşehri district of Sivas to determine the fruit quality characteristics of Uzun variety grafted onto *P. terebinthus* rootstock. Considering these results, it can be said that the protein content in the study was high and low. The differences between the protein amounts can be explained by the effect of climate conditions. In fact, Seferoglu et al. (2006) and Okay (2002) reported that protein amounts may vary depending on the region. Pistachio fruits are a rich source of oil and contain fatty acids necessary for human nutrition. The oil content in pistachios may vary among varieties and within each variety (Agar et al. 1995). Environmental conditions as well as genetic factors affect the oil content (Seferoglu et al. 2006). Seferoglu et al. (2006) reported that the oil content in their study with the long variety in 7 different regions of the Büyük Menderes basin was 46.8%–66.5%; Kuru and Ozsabuncuoglu (1990) reported that the oil content in pistachios in Türkiye was between 54% and 60%; Agar et al. (1995), who stated that the oil content of the same variety was significantly affected by different ecological conditions, reported that the oil content in pistachios varied between 48.5% and 58.5%. In the study, the oil ratio was determined as 45.20% to 51.80%, which is consistent with the results of this study. In addition, fatty acids such as hexadecanoic acid, 9‐hexadecenoic acid, methyl 8 heptadecenoate, 9‐octadecenoic acid, 9,12‐octadecadienoic acid, 9,12,15‐octadecatrienoic acid, eicosanoic acid, cis‐11‐eicosenoic acid, benzenepropanoic acid and nonanoic acid were detected in pistachio fruits. Fabani et al. (2013) reported that potassium is the most abundant element in pistachios, followed by calcium and magnesium, and trace elements are listed as Na > Fe > Zn > Cu > manganese. In the study, the mineral content was determined as follows, which is relatively consistent with these results: As a result of the analyses, nutrients such as N, Mg, Ca, K, Na, Cu, Mn, Al, Fe and Zn were detected in the fruit. When plant nutrients are compared, potassium is the most abundant in the plant, followed by Ca, Mg, Na, Fe, Zn, Al, Cu and Mn. Yang et al. (2009) reported that the order of mineral substances in pistachios is similar in terms of content. However, mineral substance concentrations may vary depending on the region, the variety used (Anderson and Smith 2005) and maintenance conditions.

## 5 Conclusion

This study revealed that different pistachio genotypes showed significant differences in terms of properties such as fruit size, yield, mineral content, oil, and protein ratios. While the G5 genotype had the largest values in terms of fruit size, the G9 genotype produced the smallest fruits. When the yield ratios of the genotypes were examined, it was determined that the G6 genotype had the highest internal yield with 46.63% and stood out in this respect. At the same time, the G6 genotype offers significant advantages in terms of storage durability with the lowest moisture content (9.10%) and high cracking rate (41.88%). While the G9 genotype was the leader in terms of protein content with 28.49%, the highest oil content with 51.80% was recorded in the 13HZN3 genotype. Significant differences were observed in terms of mineral contents among genotypes. K stood out as the mineral found in the highest amount among genotypes, and the G3 genotype had a rich content in terms of minerals such as K, Ca, Mg and Mn. Among the nutrients, Na reached high values in the G6 genotype, Al in the G5 genotype and Cu in the G10 genotype. Fatty acid analysis showed that oleic acid was the most abundant fatty acid among genotypes and the G3 genotype had the highest oleic acid content with 66.7%. However, the G10 genotype stands out in terms of rare fatty acids such as linolenic acid and pelargonic acid.

As a result, it has been revealed that there are significant differences in the physical, chemical, and biological properties of pistachio genotypes. While the G6 genotype stands out with its general performance, it has been observed that other genotypes provide superiority in certain properties such as protein ratio, fatty acid profile, or mineral content. This situation shows that genotypes should be selected according to specific growing and usage purposes. Research findings may contribute to the development of genotype‐based strategies to increase quality and yield in pistachio production.

## Author Contributions

**Umut Ates:** conceptualization, methodology, validation. **Burhan Ozturk:** writing – review and editing, writing – original draft, project administration, conceptualization, methodology. **Cuneyt Uyak:** conceptualization, writing – original draft, validation, visualization. **Adnan Dogan:** visualization, validation, resources, data curation. **Davut Alan:** conceptualization, methodology, resources. **Erdal Aglar:** writing – original draft, writing – review and editing, validation, methodology, project administration. **Orhan Durmaz:** methodology, resources, conceptualization, investigation. **Onur Tekin:** data curation, methodology, validation, visualization.

## Funding

The work was supported by the Open Access Fund of the Scientific and Technological Research Council (TÜBİTAK) of Türkiye and the Yuzuncu Yil University Scientific Research Project Department (FBA‐2023‐10364).

## Ethics Statement

Plant materials were taken from growers' orchard in Bitlis provinces. Necessary permissions were obtained verbally from the breeders for the use of the material. There is no problem in terms of ethics. In this sense, researchers are responsible for any problems that may occur and provide assurance.

## Consent

The authors have nothing to report.

## Conflicts of Interest

The authors declare no conflicts of interest.

## Data Availability

All data generated or analyzed during this study are included in this published article.
